# Conference on neuroscience and pragmatism: Productive prospects

**DOI:** 10.1186/1747-5341-6-14

**Published:** 2011-07-08

**Authors:** John R Shook

**Affiliations:** 1 Philosophy and Science Education, University at Buffalo, NY, USA

## Abstract

The conference "Neuroscience and Pragmatism: Productive Prospects" was held on June 10, 2011 at the Potomac Institute for Policy Studies in Arlington, Virginia.

## Conference Report

The conference "Neuroscience and Pragmatism: Productive Prospects" was held on June 10, 2011 at the Potomac Institute for Policy Studies in Arlington, Virginia. The program was organized by John Shook (Buffalo) and Tibor Solymosi (Southern Illinois). James Giordano, Director of the Center for Neurotechnology Studies and Vice President for Academic Programs at the Potomac Institute, was the conference co-chair and host. The conference was supported by The Society of Philosophers in America, the American Philosophical Association, and the Center for Neurotechnology Studies of the Potomac Institute for Policy Studies.

This conference brought together some pragmatist philosophers interested in neuroscience and some neuroscientists interested in pragmatism. There was much audience participation, including Q&A after each presentation, discussion through the luncheon, and audience discussion with a panel of speakers for an extended conversation. The speakers were: William Casebeer (cognitive science, Defense Advanced Research Projects Agency-DARPA, USA); Anthony Chemero (neurophilosophy, Franklin and Marshall College, PA, USA); David Franks (sociology, Virginia Commonwealth University, VA, USA); James Giordano (neuroscience and neuroethics, Center for Neurotechnology Studies of the Potomac Institute for Policy Studies, Arlington, VA, USA, and Oxford Centre for Neuroethics, University of Oxford, UK); Teed Rockwell (cognitive science, Sonoma State University, CA, USA); Jay Schulkin (biophysics and cognitive science, Georgetown University, Washington, DC, USA); John Shook (neuroethics and pragmatism, University at Buffalo, NY, USA); Tibor Solymosi (neurophilosophy, Southern Illinois University at Carbondale, IL, USA).

The speakers reminded the audience that America's first generation of pragmatists designed its theories about experience, mind, and knowledge in light of the discoveries of biology and psychology during their era. Viewing organisms entirely naturalistically and understanding human brains in light of evolution led pragmatists to radically re-conceive how intelligence works. The pragmatists were America's first cognitive scientists. Charles Peirce, William James, John Dewey, and George Mead knew the laboratory intimately and published in experimental psychology and sociology. They possessed few details on the specific functions of the brain's many components, but they were innocent of errors which later philosophers perpetuated. For example, the pragmatists noticed how the brain grows and changes in youth and they figured that the brain grew new tissue and modified its interconnections throughout the lifetime. They assumed correctly the later Hebbian idea that brain tissues activated together would be more likely to stay functionally related thereafter. They inferred that the expansion of experience and the growth of brain structure was correlated, explaining how learning consists of acquisition of workable habits for practical success in managing environing conditions. They judged that much of the brain's work occurs at non-conscious levels. They denied that cognition consists entirely of internal representations about static external matters rationalistically manipulated within a Cartesian theater, and they denied that perception, cognition, emotion, and volition were discrete processes. Their psychologies, while mildly behavioristic, were always socially oriented, emphasizing how most distinctly human capacities from language to science depend on social training, extended cognition, experimental problem-solving, and cultural accumulation of knowledge. Outspoken on knowledge and truth, their varieties of pragmatism claimed that only social groups set working criteria for truth and produce knowledge from coordinated experimental inquiry.

Pragmatism never dominated philosophy and was mostly discarded by later successive positivisms and rationalisms, but its social behaviorism and evolutionary anthropology filtered into many of the social sciences. Experimental psychology and cognitive science then rediscovered many pragmatist views of brain cognition and learning in the 1980s and 90s, and the first decade of the 21st century accelerated this trend back to pragmatism. Since active scientists are less concerned with the history or philosophical aspects of their particular fields, it comes as no surprise that most brain scientists are presently unaware of pragmatism.

William Casebeer took up the question, "What is Neuropragmatism? Some Principles and Why They Matter." He addressed some specific ways that findings of the contemporary neurosciences support a form of pragmatism, especially linking with views of John Dewey and Charles Peirce. Neuroscience is providing some insight into what is means to be an evolved, embodied organism coping with the adaptive demands of life. Such guiding principles as "Abduction come naturally," "Action is prior to representation," "Fast and frugal is fine," and "Building models is better than finding laws" shape the right account of how human intelligence works. These principles additionally highlight how pragmatic naturalism can be made consistent with our epistemic and moral norms without doing undue violence to our intuitions about either.

Teed Rockwell continued this neuropragmatist theme with his presentation on "How Computational Neuroscience Revealed that the Pragmatists Were Right." Rockwell first discussed the work of Paul Churchland, who has rejected pragmatism yet he does refer to himself as a "closet pragmatist." The pragmatism he rejects, however, is a straw slogan that bears little resemblance to the rich and complex philosophy of the original pragmatists. Furthermore, much of Churchland's best work provides both empirical justification and philosophical clarification of the epistemology of classical pragmatism. Rockwell criticized Churchland's attempt to distance himself from pragmatism while praising both Churchland's unique brand of pragmatism, and the original pragmatism of James and Dewey for which Churchland provides fresh scientific support. Since successful behavioral coordination always has cognitive aspects, unnecessary isolation of cognition from conduct is neither warranted by brain evidence or by evolution. Unscientific notions about cognition restrained by artificial philosophical concepts like "narrow content," semantic state spaces," and "mind-brain identity" obstruct neuroscience's trend towards broad content, externalism, anti-representationalism, and radical empiricism. Computational representationism relies on extreme brain modularity and functional portability (mechanical modes) but organic brains don't have such features. Only learned habitual activity can connect a person's language to the world, and trained dynamical systems can only approximate truth, as Churchland himself admits.

Like Rockwell, Anthony Chemero defended extended cognition, announcing "The End of the Debate over Extended Cognition." The philosophical debate over extended cognition has one clear point of consensus: whether cognitive systems sometimes encompass portions of the environment is an empirical question. This suggests that a clear laboratory demonstration of an extended cognitive system should end the debate once and for all. Chemero explained a series of laboratory demonstrations of extended human-tool and human-human cognitive systems, culminating in a set of experiments designed explicitly to test the thesis of extended cognition. Those tests decisively favor Gibson's theory of environmental affordances and the reality of extended cognition, so if the debate over extended cognition really is empirical, it should be over. Moreover, the empirical evidence strongly suggests that whenever cognition is extended, so too is conscious experience. However, Chemero pointed out that the debate will continue, because deniers of extended cognition simply re-define "cognition" as metaphysically "internal" and possessing "intrinsic content" in order to avoid empirical refutation.

Jay Schulkin covered wide territory in his talk on "Pragmatism, Naturalism, and the Brain." The classical pragmatists (James, Peirce, Dewey, Mead) understood some fundamental things about action, inquiry, and the brain. Their goal, a modern one, was an understanding of cognitive adaptation and learning, along with what Dewey called "lived experience," in the context of evolution of the brain. We now know that cognitive systems are not simply cortical but traverse all regions of the brain, something Dewey suggested in his 1896 paper "The Reflex Arc Concept in Psychology." Any biologically grounded view of brain function understands that bodily sensibility is essential for the diverse ways in which we explore the world. We are oriented to kinds of objects in a world that we are trying to understand. Minds inhabit bodies in cephalic systems that explore the world, and cognitive systems are embodied in real life events -- the stuff of adaptation, long noted by Darwin and James and incorporated by Dewey. A core feature of the pragmatist tradition is the embodiment of problem solving in the mind/brain. All forms of dualism are undermined by the pragmatists. Indeed, cognitive/behavioral capacities are ways to engage the world, to compute probability and assess, for example, friendly or non-friendly social events. We come prepared by neural circuits to respond to kinds of objects. Diverse cognitive systems (e.g. recognition of animate/inanimate objects, agents, senses of space, time and statistical relationships) underlie what Peirce called "abduction" or the genesis of ideas and problem solving. These biologically-derived cognitive systems are not divorced from action or perception, but are endemic to them and are distributed across neural networks in the brain.

Tibor Solymosi spoke on "Reconstruction in and of Neurophilosophy," expanding neuropragmatism's relevance to social and cultural issues. Pragmatism has resurged explicitly in neopragmatism and implicitly in neurophilosophy. Neopragmatism has focused on achieving ideals like freedom without much concern for experimental science. Neurophilosophy has had great interest in bringing experimental science to bear on philosophical problems like free will. In doing so, however, ideals like freedom are often eliminated or dismissed. Neuropragmatism cuts across these different points of emphasis by returning to what Dewey called the method of intelligence, that sees the knowledge provided by the sciences as the means for achieving our larger aims of life. The pragmatist project of reconstruction provides us with the tools to change philosophical and scientific questions like "do we have free will?" to "how does and how could freedom work?" In this way, the distinctively human way of living meaningful and value-driven lives are brought within the worldview of scientific naturalism.

David Franks's presentation centered on "Neurosociology and Some Confirmations of Chicago Pragmatism via Work on Mirror Neurons." He described how current research into mirror neurons confirms important aspects of the pragmatic tradition developed by John Dewey, George Mead and other philosophers at the University of Chicago in the 1920s. More specifically, findings from hard-nosed empirical research confirms the priority they placed on behavior, especially socially-oriented behavior. Mead's theory of the act parallels the findings of neuroscience, especially by defining objects in the environment in terms of the possibilities they hold for human action. Mirror neurons fire not only when we see others do something but they actually simulate this behavior on our motor cortex. Franks outlined some further research on implications of this theory of the social act for the ways that language is possible for humans.

John Shook's talk was on "The Emergence of Morality and the Social Self." As sociological and psychological research is demonstrating, morality as practiced by humans infrequently rises to the ideal standards set by ethical theories. Rather than permit abstract theory to first define morality, it more reasonable to investigate how actual people conduct their moral lives, where morality functions for managing interpersonal responsibilities and social relations across large social groups. Why is morality different than kinship bonds yet still restrained by relatively local familiarity? Proto-morality in early hominid species involved kinship emotions like love and social capacities such as compassion and trust. As *homo sapiens *began living in much larger tribal groups, the peculiar features of morality emerged to manage social interactions among tribal non-kin as well. Morality proper consists of generic obligations of non-maleficence and civil trust to members of one's in-group to moderate competition and permit stable cooperation across lifetimes. Morality is embodied, situated, role-embedded, habitual, cooperative, and culturally objective. Humans are capable of morality because we use social cognition and collective intentionality to actively control and manage ongoing modes of social interactions. Only the relatively recent rise of nations and empires did ethical thinking emerge under cultural pressures to deal with social interactions among people lacking common tribal/ethnic heritages and moralities. Ethics proper generates notions of abstract principles applied to generic people at all times and places, but these principles transcend the proper function of morality while uneasily co-existing along morality.

James Giordano concluded the conference by pulling together some neuropragmatist and neuroethical themes in his talk on "What 'Neuro' Really Means: Obligations for Intellectual Honesty and Veracity in Neuroethics." Ethics is the study of the processes and basis of moral decision-making. Neuroethics applies neuroscience to study the processes of moral cognition, and neuroethics is in a disciplinary position to help address the wider ethical, legal, and social issues arising from improved knowledge of brain functioning. He warned us about dangers of "neuro-ubiquity" - where adding the 'neuro' prefix to just about everything else either adds nothing, or adds reductionistic overtones that may represent ampliative arguments that are based upon rather loose and/or value-laden interpretations of neuroscientific research and findings. Properly prefixed, the 'neuro' reminds us of the brain's centrality to everything we do, of the brain's many complexities requiring multiple levels of analysis, and of the iterative and contingent nature of any neuroscientific information. Still, however, Giordano stressed that neuroscience can, and will, change our conceptions of human being and our possibilities - but we confront novel questions about balancing what we know with what we may want to make possible. He argued that the use of neuroscientific information and novel neurotechnologies will call for a practical neuroethics not bound by traditional ethics or any simplistic precautionary principle.

This conference achieved an impressively new level of interdisciplinary understanding of achieved results and set stages for further investigations. The question of "Why pragmatism now?" was not ignored. If the behavioral, cognitive, and neurosciences were only achieving new brain insights with confirmed research, any coincidence with some history of philosophical psychology would be just that, mere history. However, the broad tradition of pragmatism is needed now, more than ever. As each of the speakers took occasion to point out in different ways, knowledge of brain function rarely stays within narrow scientific bounds, nor does it stay free from philosophical interference.

Experts from neuroscience, cognitive science, and psychology are publishing their expansive judgments on ethical, legal, and social matters ranging from mental health and criminology all the way to philosophically resolving the mind-body and free will problems in any number of diverse ways. Scientists should participate in such judgments, just as the classical pragmatists did, and current scientists can benefit from their earlier philosophical explorations across the same territory. But those explorations always meet staunch resistance. Rival philosophical views, some disguised within scientific paradigms and other openly espoused by armchair philosophers, offer up staunch opposition to the new pragmatist stances all over again. Some speakers went so far as to propose that "neuropragmatism" is the kind of neurophilosophy that most fruitfully engages larger cultural questions by adequately dealing with meaningful values and ethical ideals. At the very least, the classical pragmatists are quite relevant to today's concerns at the intersection of science and culture, and a refreshed and re-tooled pragmatism will prove useful as well.

## Competing interests

The author declares that he has no competing interests.

